# Somatic Genome Variations: First Steps Towards a Deeper Understanding of an Underappreciated Source of Biodiversity and Disease

**DOI:** 10.2174/138920210793175976

**Published:** 2010-09

**Authors:** Yuri B. Yurov, Ivan Y. Iourov

Somatic Genome Variations (SGV) are referred to as intercellular variability of genomes in somatic tissues of the same organism. These can manifest as single-nucleotide changes, short DNA sequence changes (<1kb), short tandem repeat variations, retrotransposition of mobile genome elements (i.e. SINE and LINE), copy number variations and subchromosomal structural abnormalities (microdeletions, microduplications, inversions; >1kb), structural chromosome abnormalities observed at microscopic level (>2-5 Mb), alterations to chromosome morphology (i.e. fragile sites), aneuploidy (gain/loss of whole chromosomes), polyploidy (gain of haploid chromosome sets). Several lines of evidences have been reported that SGV can play a role in human biodiversity and disease. For instance, it is generally recognized that somatic genome changes produced by genomic and chromosomal instability are cancer-causing. However, related phenomena are rarely addressed in non-malignant tissues. Current genomics essentially uses technologies which operate with DNA isolated from a large pool of cells and analyzes interindividual genomic variations, whereas single-cell genomic approaches are much more rarely applied. This probably explains why SGV are significantly less appreciated. Nevertheless, previous biomedical research does provide initial data that (i) SGV appear to be widespread in human cell populations; (ii) intercellular genomic diversity seems to be associated with a number of neurological, psychiatric and immune diseases, chromosomal syndromes and cancers as well as appear to be involved in critical biological processes (intrauterine development, cell number regulation and aging); (iii) molecular cytogenetics does provide technical solutions for studying single-cell genome variations at molecular resolutions. Therefore, a need appears to exist for additional attention to an underappreciated area of single-cell genomics aimed at studying SGV.

The intention of this special issue of *Current Genomics* (Hot Topic Issue on SGV) is to gather the knowledge about causes and consequences of SGV by addressing the experience of leading experts in fields of human genetics, genomics and molecular cytogenetics. This attempt appears to be successful, inasmuch as the line of reviews has provided for an integrated view of how SGV can be involved in human interindividual diversity, normal prenatal development, aging and pathological changes associated with a number of diseases. The issue begins with theoretical considerations about possible phenotypic effects of SGV and about inevitable changes of current concepts in genomics (epigenomics) resulting from research of SGV. Then, a brief overview of SGV in health and disease is given. The issue continues with two articles dedicated to SGV during human prenatal development. The first one describes recent data on intercellular genomic changes in early human embryos and suggests possible effects at further prenatal developmental stages. The second one gives a timely overview of SGV in extra-embryonic tissues and provides convincing evidence that these do play a significant role in the normal placentation. The next article presents an original hypothesis suggesting one of the most common genetic abnormalities in human newborns (trisomy 21) to be a result of intercellular genomic variations in fetal tissues. Furthermore, an extensive overview of SGV manifesting as aneuploidy involving chromosome 21, which are associated with a broad array of diseases, is given. To end the description of SGV in human embryonic and fetal tissues, a review of ontogenetic genome variations is provided. Surveying data on intercellular genome variability from conception to late ontogeny, it was possible to show that SGV are involved in controlling cell numbers during development and aging. Additionally, a phylogenetic model of “dynamic genome” was adapted to cell populations suggesting similar genetic processes to take part as during phylogeny as during ontogeny. Further, the origin of genetic mosaicism produced by SGV manifesting as copy number variations (one of the most common type of genomic variations) is described. According to authors’ data and to the literature, this type of SGV is likely to from during embryonic development remaining stable (cell proportions) as long as 20 years. Continuing evaluations of SGV in liveborns, a review of mosaic small supernumerary marker chromosomes, which represent a frequent type of chromosome abnormalities, is presented and the importance of such cases for prenatal diagnosis is underlined. Diagnostic problems related to SGV and possible ways of their solutions are further described in the next review. Here, an overview of genomic and chromosomal instabilities as well as literature data on identification of SGV has allowed to come to an optimistic conclusion that it is possible to propose recommendations on molecular cytogenetic diagnosis and clinical interpretation of SGV. Focusing on medical aspects, it would be interesting to evaluate SGV in a certain disease. This task was successfully completed by a review addressing genomic instabilities in schizophrenia. Finally, a review showing the potential of modeling SGV (somatic copy number variation) and germline genomic variation for biomedical research is presented.

All together, the articles in this Hot Topic Issue provide an exciting review of current SGV research that can stimulate readers to pay more attention to single-cell and somatic cell genomics forming a basis for further studies in this area of genomics and epigenomics.

This special issue of Current Genomics is dedicated to the memory of our close relative and colleague, Ilia V Soloviev, a talented young researcher and a pioneer of molecular cytogenetics, genome and chromosome research, whose prodigious work has formed our current research directions.

